# The importance of formal versus informal mindfulness practice for enhancing psychological wellbeing and study engagement in a medical student cohort with a 5-week mindfulness-based lifestyle program

**DOI:** 10.1371/journal.pone.0258999

**Published:** 2021-10-21

**Authors:** Naomi Kakoschke, Craig Hassed, Richard Chambers, Kevin Lee

**Affiliations:** 1 School of Psychological Sciences and Turner Institute for Brain and Mental Health, Monash University, Melbourne, Australia; 2 Health & Biosecurity, CSIRO, Adelaide, South Australia, Australia; 3 Department of General Practice, Monash University, Melbourne, Australia; 4 Centre for Consciousness and Contemplative Studies, Monash University, Melbourne, Australia; 5 Campus Community Division, Monash University, Melbourne, Australia; 6 Department of Medicine, Monash University, Melbourne, Australia; 7 Department of Physiology, Monash University, Melbourne, Australia; Sunway University, MALAYSIA

## Abstract

**Purpose:**

Medical students commonly experience elevated psychological stress and poor mental health. To improve psychological wellbeing, a 5-week mindfulness-based lifestyle course was delivered to a first-year undergraduate medical student cohort as part of the core curriculum. This study investigated the effects of the program on mental health, perceived stress, study engagement, dispositional mindfulness, and whether any improvements were related to amount of formal and/or informal mindfulness practice.

**Methods:**

Participants were first year undergraduate medical students (N = 310, 60% female, *M* = 18.60 years) with N = 205 individuals completing pre and post course questionnaires in a 5-week mindfulness-based lifestyle intervention. At pre- and post-intervention, participants completed the Mental Health Continuum-Short Form, the Perceived Stress Scale, the Utrecht Work Engagement Scale for Students, the Freiburg Mindfulness Inventory, and the Mindfulness Adherence Questionnaire.

**Results:**

Mental health, perceived stress, study engagement, and mindfulness all improved from pre- to post-intervention (all *p* values < .001). Improvements on these outcome measures were inter-related such that PSS change scores were negatively correlated with all other change scores, FMI change scores were positively correlated with MHC-SF and UWES-S change scores, the latter of which was positively correlated with MHC-SF change scores (all *p* values < .01). Finally, observed improvements in all of these outcomes were positively related to informal practice quality while improved FMI scores were related to formal practice (all *p* values < .05).

**Conclusions:**

A 5-week mindfulness-based program correlates with improving psychological wellbeing and study engagement in medical students. These improvements particularly occur when students engage in informal mindfulness practice compared to formal practice.

## Introduction

It is well documented that medical students experience higher levels of psychological distress and have poorer mental health, including increased rates of depression, suicidal ideation, and substance use disorder relative to non-medical students [1–5]. A recent systematic review found wide ranging prevalence estimates of 7.7–65.5% for anxiety, 6.0–66.5% for depression and 12.2–96.7% for psychological distress [6], with another meta-analysis finding that 11.1% experienced suicidal ideation with only 15.7% of medical students with depression seeking psychiatric treatment [7].

Despite having similar or better mental wellbeing prior to commencing medical training, the mental health of medical students declines significantly throughout their training [8, 9]. Distress in medical students has been shown to develop during the first year of training across multiple domains of psychological distress, including poorer quality of life, higher fatigue, and burnout. The prevalence is alarmingly high with estimates showing that 50% of 2000 medical students surveyed experienced multiple forms of distress and had thoughts of dropping out, which increased the risk of suicidal ideation by 15 times relative to those without any reported distress [10].

If distress experienced during medical training progresses to chronic occupational stress when medical students graduate, this can become a major contributor to professional burnout. Professional burnout is well documented among both medical students and doctors with prevalence rates among interns estimated to be 75% [11–13]. Similarly, a recent meta-analytic review found that one in two medical students was estimated to experience burnout prior to undertaking residency [14]. Importantly, psychological distress and burnout amongst health care professionals can have serious implications for patients including less compassionate medical care and more prescription errors [12, 15, 16]. Therefore, it is important to investigate scalable prevention strategies to reduce distress at an early career stage in order to prevent academic and professional burnout and potentially improve clinical performance and patient care.

One promising strategy for reducing psychological distress in clinicians is mindfulness-based interventions [17, 18]. Such interventions aim to improve trait mindfulness, which refers to regulating attention and being present in the moment as well as cultivating a non-judgmental, accepting, and compassionate attitude to one’s moment-by-moment experience. This state of awareness applies to both internal physical and psychological states [19, 20].

Mindfulness training has been shown to reduce distress through earlier recognition of stress responses, increased self-awareness of thoughts and reduced rumination [21]. Recent meta-analyses have found that mindfulness-based interventions can improve stress, depression, burnout, and fatigue in medical students [22]. Mindfulness interventions have also been shown to reduce burnout and improve wellbeing in health-care professionals and trainees [23]. Nevertheless, given the variability of mindfulness-based programs and uncertainty about which program components lead to improvements, it is important to test the efficacy of such programs for improving psychological health among medical students. Importantly, these programs are low-cost as well as easily implementable. Thus, it makes sense to explore ways of delivering mindfulness training as early as possible including integrating such programs into existing first-year medical curriculum.

Generally, the practice of mindfulness can be practised formally or informally. Formal practice refers to meditation of which there are several forms [24], while informal practice refers to being mindful outside of meditation while undertaking daily activities such as eating [25] and walking [26]. Different mindfulness teachers and programs differentially emphasise the importance of these two aspects of practicing mindfulness, but it is not yet known whether formal or informal practice is more strongly associated with positive outcomes nor whether this differs between different cohorts. The few studies that have measured differences in formal versus informal mindfulness practice have mostly been conducted in clinical settings and have produced conflicting results [27–29]. Thus, it is important to consider whether formal or informal mindfulness practice is more appropriate when delivering mindfulness training as a part of core-curriculum, i.e., a compulsory rather than an opt-in part of the curriculum.

Regardless of differences in formal or informal mindfulness practice, previous research clearly shows that health outcomes have a stronger association with amount of actual mindfulness meditation practice, rather than the length of sessions or duration of the program itself [30, 31]. Furthermore, how well mindfulness is received and practiced is likely dependant on how effectively mindfulness is integrated, contextualised and assessed within the curriculum [32]. Thus, it is important to consider medical students’ level of adherence to mindfulness practice, formal and informal, when evaluating the effectiveness of mindfulness-based programs delivered within a university context [33].

### The current study

Since 2002, the medical school at Monash University (Melbourne, Australia) has integrated mindfulness-based programs into the core curriculum [32, 34]. This program has previously shown promising results including improved dispositional mindfulness and study engagement [35] as well as reduced anxiety, stress and depression even during critical times such as the pre-exam period [19], in addition to improvements in self-care and psychological distress [36].

The primary aim of the current study was to examine whether a 5-week curriculum-based mindfulness program improved mental health, psychological stress, dispositional mindfulness, and study engagement in first year medical students. A secondary aim of the study was to investigate the relationships between improvements in key outcome measures (i.e., mindfulness, perceived stress, mental health, and study engagement). Finally, we aimed to examine whether adherence to formal and informal mindfulness practices were associated with improvements in these outcomes.

First, we hypothesised that perceived stress, study engagement, mindfulness, and mental health would improve over the course of the 5-week program. Second, we hypothesised that improvements in stress, engagement, mindfulness and mental health would be positively correlated with each other. Third, we hypothesised that greater adherence to daily practice (i.e., self-directed formal and informal mindfulness practice) would be negatively correlated with changes in stress and positively correlated with changes in study engagement, mental health, and mindfulness.

## Materials and methods

### Participants

Being a compulsory component of first year medical course, participants consisted of all first-year medical students N = 310 comprising of a preponderance of females (n = 134; 60%) with a mean age of 18.6 years. Of the students in the course, 66.1% (n = 205) completed both pre- and post-course questionnaires. One of the ten tutors failed to administer the post-course questionnaires which contributed to the reduced response rate.

### Intervention

#### Mindfulness program

Following introductory lectures that examined the research behind health and wellbeing practices, a mindfulness wellbeing program consisting of five weekly sessions of 2 hours in duration was conducted. The tutorials were facilitated by health professionals with significant personal and professional experience in mindfulness (class size between 28–32 students per tutor). During these tutorials, the students learned to practice formal mindfulness meditation as well as informal practices that integrate mindfulness into their daily lives.

Students were also encouraged to engage with weekly home-based formal and informal practice and were required to submit a reflective journal each week based on their experience of, insights into, and questions about applying mindfulness in their own lives. Students discussed their practice, insights and questions each week during tutorials.

The recommended formal practice began with 5-minute meditations twice a day and shorter 1-2-minute practice sessions more regularly throughout the day. Students were also encouraged to practice mindfulness informally in daily life, for example while eating, doing chores, or walking. Four cognitive practices, namely perception, letting go, acceptance and being present, are designed to help students identify and reflect upon important cognitive aspects of mindfulness [37] were introduced, one per week. After introduction, students were instructed to apply the cognitive practice during the week and their experiences were discussed on the following week.

In addition to a focus on the wellbeing-related aspects of mindfulness, students were also explicitly encouraged to contextualise mindfulness and explore how it relates to their academic studies and development of clinical competencies. For example, students were given guidance on how to unhook from ruminative thought patterns, engage attention while studying, communicate with patients and peers more mindfully, manage exam anxiety and avoid the distracting impact of technology and the trap of complex ‘multitasking’. Previous research has found that contextualising mindfulness in this way enhances engagement [19]. The tutorials also included a series of role-plays where students were required to help a dummy patient understand stress, the mind-body relationship and the basic principles of mindfulness. The reflective journal was formatively assessed as a hurdle task because the attribution of marks to the journal would likely encourage students to write a ‘model’ journal rather than an authentic and truly reflective one. Despite core knowledge being assessable, the students’ personal choice to practice mindfulness within tutorial time or in their own personal life was entirely their choice, although past research with a similar cohort found that once students understood the background research and rationale for mindfulness, 90.5% reported personally practising and applying mindfulness in their lives [19].

### Measures

#### Perceived Stress Scale (PSS)

The PSS is a 10-item scale that measures perceived psychological stress [38]. Each item is rated on a Likert-type scale ranging from 1 (*Never*) to 5 (*Very often*). Total scores are obtained by reversing responses for the four positively phrased items (items 4, 5, 7, and 8) and then summing across all scale items with higher scores indicating higher perceived stress. The PSS has been shown to have concurrent and convergent validity and excellent internal consistency with estimates ranging from α = .84 to .90 [39–41], which was the case in the current study (pre- and post-intervention: α = .85).

#### Mental Health Continuum-Short Form (MHC-SF)

The MHC-SF is derived from the long-form scale and was used to measure wellbeing [42]. This 14-item scale comprises 3 subscales that each measure a different facet of wellbeing: emotional (3 items), psychological (6 items) and social (5 items). Respondents are asked to rate the frequency of experiencing every feeling in the past month on a Likert-type scale ranging from 1 (*never*) to 6 (*every day*). Scores for each item are summed to calculate a total score ranging from 0–70 with higher scores indicating better mental health. The MHC-SF has shown excellent discriminant validity and internal consistency (α = >.80; [42, 43]). In the current study, internal consistency was also excellent (pre-intervention: α = .91, post- intervention: α = .92).

#### Utrecht Work Engagement Scale for Students (UWES-S)

The UWES-S was used to measure student study engagement [44]. This 14-item scale comprises three subscales: 1) Vigor (5 items): mental resilience and high energy levels while studying, and one’s willingness to put effort into one’s work (e.g., *When I get up in the morning I feel like going to class*); 2) Dedication (5 items): a sense of involvement, significance, inspiration, challenge, enthusiasm, and pride (e.g., *I find my studies to be full of meaning and purpose*); and 3) Absorption (4 items): high engagement with study (e.g., *I get carried away when I’m studying*). Each item is scored on a 6-point Likert-type scale ranging from 1 (*Almost never/A few times a year or less*) to 6 (*Always/Every day*) with higher scores indicating higher study engagement. The UWES-S has high content and factorial validity [44] as well as high internal consistency (α = .74 to .87) [45], which was also the case in the current study (pre- intervention: α = .87, post- intervention: α = .90).

#### Freiburg Mindfulness Inventory (FMI)

The FMI was used to measure the general construct of mindfulness comprising non-judgemental present-moment observation [46]. The scale comprises 14 items that are scored on a 4-point Likert-type scale ranging from 1 (*Rarely*) to 4 (*Almost always*) with higher scores indicating higher mindfulness. Items include statements such as: *I watch my feelings without getting lost in them*, and *I accept unpleasant experience*. The FMI has high internal reliability and is shown to measure mindfulness as distinct from other related concepts such as self-awareness (α = .86) [46]. In the current study, internal consistency was acceptable (pre-intervention: α = .79, post-intervention: α = .82).

#### Mindfulness Adherence Questionnaire (MAQ)

The MAQ was used to measure amount and quality of formal meditation and informal mindfulness practice undertaken in the preceding week [47]. The scale comprises 12 questions, two of which are ordinal questions relating to frequency and amount of meditation practice (quantity), and 10 of which ask respondents to rate the quality of their formal and informal mindfulness practice (items 1–4: formal subscale; items 5–10: informal subscale) on a 7-point Likert-type scale ranging from 0 (*Never*) to 6 (*Always*) with higher scores indicating higher mindfulness practice quality (see S1 Appendix). Practice quality refers to the level to which a person is practicing training the two main aspects of mindfulness (attention and attitude) during formal and informal practice. Items for measuring the quality of the formal practice include statements such as: “*When meditating*, *how much of the time were you practising an accepting attitude toward what you were experiencing*?*”* Items for measuring the quality of the informal practice include statements such as: *“In your daily life*, *how much of the time were you practicing bringing your attention back to what you were doing when it wandered off*?*”* The MAQ has good discriminant validity and acceptable to good internal consistency for the formal subscale (α = .67 to .87) and the informal subscale (α = .91 to .93) [47]. In the current study, internal consistency was low for the formal subscale (pre-course: α = .34, post-course: α = .33), but excellent for the informal subscale (pre-course: α = .85, post-course: α = .84).

### Procedure

At pre-intervention (T1; mid-semester), participants provided written informed consent before completing several self-report questionnaires, namely, the PSS, MHC-SF, UWES-S, FMI, and the MAQ. They then engaged in the 5-week mindfulness-based HEP. At post-intervention (T2; pre-exam week), the participants completed the above questionnaires again. Ethics approval was obtained from the Monash University Human Research Ethics Committee (MARP/2011/170). Additional permission was gained from the Monash School of Medicine to conduct research with medical students.

### Data analysis

A series of repeated measures ANOVAs were conducted to examine the effect of the 5-week mindfulness program on each of the key outcomes, namely, stress, mental health, study engagement, and mindfulness (as measured by the PSS, MHC-SF, UWES-S, and FMI). Change scores (i.e., T2-T1) were calculated for the PSS, UWES-S, MHC-SF and FMI as well as for the MAQ. Pearson product-moment correlation analyses were conducted to examine associations between change scores on each of the key outcomes (i.e., PSS, UWES-S, MHC-SF and FMI). Pearson product-moment correlational analyses were conducted to investigate associations between change scores on each of the outcomes with MAQ scores, including the mean weekly (1) frequency of meditation sessions, (2) duration of each session (in minutes), and (3) quality of formal and informal meditation practice. Finally, as an exploratory analysis, a series of one-way ANOVAS were conducted to examine whether mindfulness practice adherence level predicted changes in the outcomes. We divided the sample into tertiles based on MAQ change scores (high, moderate, and low) separately for informal and formal practice quality based on previous studies using a similar approach [48, 49]. For significant multivariate effects, follow-up post-hoc t-tests were conducted to examine differences in scores between adherence groups using Bonferroni corrections for multiple comparisons. Significance was set at *p* < .005. Data were analysed using IBM SPSS Statistics version 25 (IBM Corp., Armonk, N.Y., USA).

## Results

### Changes in PSS, FMI, UWES-S, and MHC-SF scores over time

The repeated measures ANOVA for PSS scores revealed a significant effect for time, *F*(1,195) = 21.57, *p* < .001, *η*^2^ = 0.10, due to a small decrease from T1 to T2 (see Table 1 for descriptive statistics). The repeated measures ANOVA for FMI scores revealed a significant effect for time, *F*(1,194) = 82.67, *p* < .001, *η*^2^ = 0.30, due to a moderate increase from T1 to T2. The repeated measures ANOVA for UWES-S scores revealed a significant effect for time, *F*(1,195) = 63.73, *p* < .001, *η*^2^ = 0.25, due to a moderate increase from T1 to T2. Finally, the repeated measures ANOVA for MHC-SF scores revealed a significant effect for time, *F*(1,194) = 26.18, *p* < .001, *η*^2^ = 0.12, due to a small increase from T1 to T2. Overall, these findings indicate that the HEP program improved participants’ levels of stress, mindfulness, study engagement and mental health over time.

**Table 1:** Descriptive statistics for PSS, FMI, UWES-S, and MHC-SF scores at pre- and post-intervention.

Variable	Pre-Intervention		Post-Intervention		
	*M*	*SD*	*M*	*SD*	*d*
PSS	28.20	5.59	26.70	5.40	0.33
FMI	34.79	6.01	38.55	6.07	0.65
UWES-S	3.67	0.76	3.98	0.83	0.56
MHC-SF	45.28	11.37	48.48	11.34	0.36

*Note*: M = mean, SD = standard deviation, *d* = Cohen’s d, PSS = Perceived Stress Scale, FMI = Freiburg Mindfulness Inventory, UWES-S = Utrecht Work Engagement Scale-Student Form, MHC-SF = Mental Health Continuum Short Form.

### Associations between PSS, FMI, UWES-S, and MHC-SF change scores

Results showed that PSS change scores were significantly negatively correlated with all other change scores, while FMI change scores were significantly positively correlated with both MHC-SF and UWES-S change scores, the latter of which was also positively correlated with MHC-SF change scores (see Table 2 for correlation coefficients). The results indicate that reductions in stress were related to improvements in mindfulness, study engagement, and mental health, while improvements in mindfulness were associated with improvements in study engagement and mental health, and improvements in the latter were associated with improvements in study engagement.

**Table 2:** Pearson product-moment correlations for PSS, FMI, UEWS-S, and MHC-SF change scores.

Variable	1.	2.	3.	4.
1. PSS		-.37**	-.36**	-.45**
2. FMI			.34**	.46**
3. UEWS-S				.26**
4. MHC-SF				

Note: ** significant at the 0.01 level (2-tailed). PSS = Perceived Stress Scale, FMI = Freiburg Mindfulness Inventory, UWES-S = Utrecht Work Engagement Scale-Student Form, MHC-SF = Mental Health Continuum Short Form.

### Associations between PSS, FMI, UWES-S, and MHC-SF change scores and MAQ scores

Change scores on the PSS, FMI, UWES-S, and MHC-SF and were positively correlated with improvements in quality of informal practice, while improved FMI scores were positively correlated with an increased number of meditation sessions (see Table 3 for correlation coefficients). In other words, the results indicate that students improved more on all of the outcome measures if informal mindfulness practice was higher in quality, and they improved more on FMI scores if they completed more formal mindfulness practices.

**Table 3:** Pearson product-moment correlations between change in outcomes and MAQ scores.

	MAQ scores			
	Practice quality		Practice Quantity	
Variable	Formal	Informal	Frequency of sessions	Minutes per session
PSS	-.16	-.23**	.01	.05
FMI	.20	.36**	.17*	.03
UEWS-S	.33	.38**	.07	.04
MHC-SF	-.02	.22**	.12	-.07

Note: ** significant at the 0.01 level (2-tailed),

* significant at the 0.05 level (2-tailed).

### Changes in PSS, FMI, UWES-S, and MHC-SF by low, moderate, and high mindfulness adherence

One-way ANOVAS revealed significant differences in study engagement, *F*(2,188) = 14.342, *p* < .001, mindfulness, *F*(2,187) = 9.238 *p* < .001, mental health, *F*(2,187) = 4.221, *p* = .016, and perceived stress change scores, *F*(2,189) = 6.650, *p* = .002, between the low, moderate and high informal practice adherence groups (see Table 4 for descriptive and inferential statistics). Post hoc tests revealed participants with higher informal practice quality adherence showed larger improvements in study engagement than those with moderate or lower informal practice adherence, while there was no difference between moderate and lower practice adherence. For mindfulness, mental health, and perceived stress, those with higher informal practice adherence showed larger improvements than those with lower informal practice adherence, while there was no difference between higher and moderate or moderate and lower informal practice adherence.

**Table 4:** Descriptive and inferential statistics for post hoc comparisons for low, medium, and high adherence to informal mindfulness practice.

	Informal Practice Quality					
	Low	Moderate	High	Low-Moderate	Low-High	Moderate-High
**Variable**	*Mean (standard deviation*)			*Mean difference (p value)*		
PSS	0.02 (3.47)	-1.77 (4.17)	-2.77 (5.28)	1.79 (.061)	2.79 (.001)	1.00 (.586)
FMI	1.67 (5.70)	3.89 (5.11)	5.92 (5.76)	-2.22 (.071)	-4.25 (< .001)	-2.03 (.123)
UWES-S	0.10 (0.54)	0.26 (0.48)	0.60 (0.56)	-0.16 (.269)	-0.49 (< .001)	-0.33 (.001)
MHC-SF	0.04 (0.55)	0.28 (0.62)	0.35 (0.67)	-0.24 (.094)	-0.30 (.019)	-0.07 (1.00)

Note: PSS = Perceived Stress Scale, FMI = Freiburg Mindfulness Inventory, UWES-S = Utrecht Work Engagement Scale-Student Form, MHC-SF = Mental Health Continuum Short Form.

For formal practice adherence, there was also a significant difference between the low, moderate and high formal practice quality groups for change in study engagement, *F*(2,32) = 4.052, *p* = .027. No other omnibus effects were significant. Post hoc tests revealed participants reporting higher formal practice adherence showed larger improvements in study engagement than those with lower formal practice adherence (*M* = .44, *SD* = .41 versus *M* = .01, *SD* = .44, mean difference = -.44, *p* = .023), while there was no significant difference between higher and moderate (*M* = .44, *SD* = .41 versus *M* = .22, *SD* = .24, mean difference = -.22, *p* = .540) or moderate and lower (*M* = .22, *SD* = .24 versus *M* = .01, *SD* = .44, mean difference = -.22, *p* = .587) formal practice adherence.

## Discussion

This study examined the relative contribution of formal and informal mindfulness practice to psychological improvements resulting from a 5-week mindfulness course [50]. The mindfulness course in this study was a compulsory part of the university curriculum delivered to a cohort of medical students known to experience high levels of stress. The psychological outcomes examined included both positive and negative measures, namely, study engagement, mental health, mindfulness, and perceived psychological stress.

In accordance with our first hypothesis, participants reported a reduction in perceived stress, as determined by the Perceived Stress Scale (PSS). Prior to the program, the students’ overall PSS scores indicated they had high levels of stress, whereas after the program their scores had significantly reduced to moderate levels. Though an improvement is important, its relationship to the mindfulness course remains a hypothesis as it is difficult to prove without a control group, an aspect of the study which is discussed later in terms of limitations. Nevertheless, prior randomized control trials of mindfulness-based programs have generally shown a positive effect on PSS scores [17, 51].

Importantly, participants also demonstrated improvements on all other key outcomes, including dispositional mindfulness as indicated by the Freiburg Mindfulness Inventory (FMI), study engagement as determined by the Utrecht Work Engagement Scale for Students (UWES-S), and overall emotional, psychological, and social health as measured by the short form Mental Health Continuum (MHC-SF). The average post-intervention FMI score (38.55) was higher than the score of 37.24 observed in the non-clinical sample during the development of the FMI [46]. Though difficult to compare with other medical cohorts, the UWES-S in our cohort is encouraging with post-intervention UWES-S mean score of 3.98 compared to 3.19 observed in a study of Chinese undergraduate medical students [52]. Similarly, the MHC-SF with an average score of 48.48 observed in our study was comparable to the average score of 54.12 observed by Zemojtel-Piotrowska et al., [53] in their large-scale study across 38 nations. The relatively good mental health of our cohort is remarkable given that the post-intervention scores were measured during the typically highly stressful pre-exam period.

Perhaps the most important and unique finding from this study is that informal practice was associated with improvements in study engagement, trait mindfulness, perceived stress and overall mental health, while formal practice was only related to improved trait mindfulness. This findings adds to the small body of literature which indicates the importance of informal mindfulness practice being greater than formal practice in a quantitative manner [54, 55]. There are other high quality studies that emphasize the importance of informal practice that demonstrate this in a qualitative manner [56], which is no less important than quantitative studies. Taken together, these findings highlight the importance of informally practicing mindfulness during daily activities.

The finding that informal practice had a greater effect than formal practice in our cohort may be indicative of outcomes of programs for non-self-selected participants. In other words, where mindfulness is mandated as a part of school, university curriculum or workplace, participants may have less motivation to practice formal aspects of mindfulness like mindfulness meditation regularly, but may be more willing to conscientiously apply mindfulness informally in their lives if it can be made relevant and contextualised for them. It may also be that participants engaged in ‘opt in’ mindfulness studies self-select and are at a stage where formal practices are more appealing.

Importantly for a university program, we showed that study engagement improved. This is notable, given that most medical students are already highly conscientious, but became even more so after the mindfulness training. Moreover, study engagement and work engagement are identical constructs (albeit in different contexts), and the latter has been associated with reduced burnout and subsequent better quality care amongst clinicians working in a high pressure environments such as intensive care units [57]. The model proposed by Schaufeli et al. (2002), which underpins the UWES-S used in this study, indicates that there are three aspects of work/study engagement. The first is ‘Vigour’, which refers to being energised by work/study and remaining resilient. The second is ‘Dedication’, which refers to being highly involved in and feeling challenged, enthusiastic and inspired by work/study. The third is ‘Absorption’, which refers to being engrossed in work/study. Not only did the overall UWES-S show improvement, but all three subscales of the UWES-S showed improvements.

A key concern of medical educators is how to help medical students enter a workforce that has a high rate of burnout typified by exhaustion or feeling emotionally worn out, depersonalised with patients, and a sense of reduced accomplishment. The abovementioned findings of improved study engagement are promising in this regard, since study/work engagement has been shown to be the opposite of burnout [58]. Previous research has also shown a negative relationship between mindfulness and burnout in health professionals (for a review see: [59]). Mindfulness includes, among other things, the ability to be more patient with oneself when things go wrong, and also to appreciate oneself in daily life. The finding in our study that change scores on the FMI were significantly associated with change scores on the UWES provides further support for this notion.

Interestingly, improvements were observed in perceived stress over the period in which the medical students were engaged in the mindfulness course and practice. This finding is particularly encouraging as previous authors have found that stress contributes to reduced overall mental health [60]. Furthermore, in the current study, the pre-intervention assessment was conducted mid-semester while the post-intervention assessment was conducted in the week prior to exams. The finding that stress *decreased* during this period is notable, as it suggests that core-curriculum mindfulness training may be a very useful addition to high-stress degrees such as medicine. Such research is especially important given that the typical contribution in terms of perceived stress toward mental health is around 20–40% [61]. Thus, we can be cautiously optimistic about the potential beneficial effects of our mindfulness course for medical students.

The observed correlational findings of this study based on questionnaires have important theoretical implications. First, results of this study indicate that a mindfulness-based course for medical students may have therapeutic effects on study engagement. Second, adherence to both formal, but more so informal mindfulness practice, appears to be associated with a stronger ability to stay present as indicated by the FMI, and this in turn may help to uncouple stress from mental health, which promotes study engagement.

The strengths of this study include the large sample size, focus on a population with high levels of stress and burnout (namely, medical students), measurement of informal practice in addition to formal meditation practice quantitatively, and the examination of a mindfulness program embedded in the curricula. Nevertheless, this study is subject to some limitations. First, as the mindfulness-based program covers other topics such as exercise and nutrition along with mindfulness, it is hard to quantify the contribution of these lifestyle factors from mindfulness to the observed improvements in our cohort as they were not measured. Second, the desirable inclusion of an active or passive wait-list control group is generally not possible in programs imbedded within the curriculum where the whole cohort receives it at the same time. The lack of a control group in the current study meant that the observed improvements cannot be directly attributed to the mindfulness-based program and remains a correlation. Although it may be a limitation, the use of a compulsory course reduces self-selection bias, such that participant characteristics are unlikely to confound effects seen and the benefit of such a program would more likely be generalizable to the population than studies where participation was voluntary. Nevertheless, compulsory programs need to emphasise safeguarding against the negative impacts of mindfulness practice which are not common but range from depersonalization, initial mood deterioration to resurfacing of past trauma [62]. The presence of experienced tutors that discussed the practice, insights and questions gained each week over five weekly small tutorial groups as well as submitted journaling as we discussed in the methods section served as safeguards.

Finally, our study design was pre-post and did not include a longer-term follow-up. Most mindfulness-based programs are limited to a relatively short-term period of less than three months [63, 64]. Nevertheless, Slonim et al. [36] followed-up cohorts of Monash University medical students across five year levels and found dispositional mindfulness to be a significant moderator of the relationship between self-care and psychological distress. Thus, future research should aim to examine the long-term effects of mindfulness training by following up with students throughout their degree and potentially supplement their ongoing mindfulness practice with smartphone applications that can be available for longer term support and facilitation of mindfulness practice [65].

A longer-term follow-up beyond university studies is also warranted to examine whether improvements in overall health are maintained during the transition into work, given that a primary purpose of mindfulness-based programs at university is to equip students with tools needed to help them manage high-pressured work once they graduate. Evidence is clear that psychosocial stress at work impacts not only mental health, but also physical health (e.g., cardiovascular disease and diabetes) over the longer-term, as demonstrated in the seminal Whitehall studies [66]. Thus, future research should aim to determine the longer-term impact of the program on both physical and mental health.

## Conclusion

In summary, this study provides evidence for the potential benefits of implementing a mindfulness-based program within the curriculum for first year medical students, including improved mental health, study engagement, and dispositional mindfulness, and reduced perception of stress over the course of the semester. This study also produced interesting preliminary findings in relation to the relative impact of both quality and quantity of mindfulness practice. Longitudinal research is needed to determine if the effects of mindfulness training are sustained over time and future studies should include active control groups to account for potential cohort effects. Overall, the results indicate that mindfulness may be a promising intervention for improving psychological health among medical students through a strong emphasis on informal mindfulness practice.

## Supporting information

S1 Appendix Mindfulness Adherence Questionnaire. (DOCX)
