# Supplementary material for: The importance of formal versus informal mindfulness practice for enhancing psychological wellbeing and study engagement in a medical student cohort with a 5-week mindfulness-based lifestyle program
Source: PLoS One. 2021 Oct 21;16(10):e0258999. doi: 10.1371/journal.pone.0258999 (PMC8530308; doi:10.1371/journal.pone.0258999)
Supplement: S1 Appendix — (DOCX) [file pone.0258999.s001.docx]

**Supplementary Information**

***Mindfulness Adherence Questionnaire***

**The MAQ is designed to measure the quantity and quality of your mindfulness practice over the past week.**

**Questions 1 to 6 ask about your formal meditation practice (e.g. sitting meditation).**

1. How many times did you do formal meditation practice in the past week? _________ times

1. What was the average duration of each meditation session? _________ minutes (please give estimate, not range)

(Note: If you didn’t do any meditation practice in the past week, please skip to question 7)

**Please tick the appropriate box (0 – “Never” to 6 – “Always”) to indicate how much each of the following were true for you over the past week.**

**When meditating, how much of the time:**

|  | 0  Never | 1  Rarely | 2  Sometimes | 3  Half the time | 4  A lot | 5  Most of the time | 6  Always |
| --- | --- | --- | --- | --- | --- | --- | --- |
| 1. was your attention focused on what you intended to focus on (body, breath, sounds etc.)? |  |  |  |  |  |  |  |
| 1. were you noticing when you were getting distracted? |  |  |  |  |  |  |  |
| 1. were you practicing an accepting attitude toward what you were experiencing? |  |  |  |  |  |  |  |
| 1. were you practicing being gentle and compassionate toward yourself? |  |  |  |  |  |  |  |

**Questions 7 to 12 ask about your informal practice (being mindful in everyday life, outside of meditation sessions).**

**Again, please tick the box to indicate how much each of the following were true for you over the past week.**

**In your daily life, how much of the time were you practicing:**

|  | 0  Never | 1  Rarely | 2  Sometimes | 3  Half the time | 4  A lot | 5  Most of the time | 6  Always |
| --- | --- | --- | --- | --- | --- | --- | --- |
| 1. paying attention to everyday activities (e.g. eating, walking, chores, communicating etc.)? |  |  |  |  |  |  |  |
| 1. paying attention while working or studying? |  |  |  |  |  |  |  |
| 1. bringing your attention back to what you were doing when it wandered off? |  |  |  |  |  |  |  |
| 1. being aware of your thoughts, emotions and reactions? |  |  |  |  |  |  |  |
| 1. bringing an accepting attitude toward what you were experiencing? |  |  |  |  |  |  |  |
| 1. being gentle and compassionate toward yourself? |  |  |  |  |  |  |  |

**Instructions:**

Questions 1 and 2 give an estimate of the *quantity* of mindfulness meditation practice in terms of frequency and duration. These can be multiplied together to give the total number of minutes practiced per week.

Questions 3 to 12 assess the *quality* of formal and informal practice.

Questions 3 to 6 measure how consistently the person practiced paying attention and cultivating a mindful attitude while meditating. Add up these items to give a sub-score for formal practice quality.

Questions 7 to 12 measure how consistently the person practiced paying attention and cultivating a mindful attitude during daily life. Add up these items to give a sub-score for informal practice quality.

Add up all items from 3 to 12 to get a total score for quality of mindfulness practice.

There are no reversed items.
